# Demonstrating Faster Multi‐Label Grey‐Level Analysis for Crack Detection in Ex Situ and Operando Micro‐CT Images of NMC Electrode

**DOI:** 10.1002/smtd.202500082

**Published:** 2025-06-23

**Authors:** Matthew P. Jones, Huw C. W. Parks, Alice V. Llewellyn, Hamish T. Reid, Chun Tan, Aaron Wade, Thomas M. M. Heenan, Francesco Iacoviello, Shashidhara Marathe, Paul R. Shearing, Rhodri Jervis

**Affiliations:** ^1^ Electrochemical Innovation Laboratory Department of Chemical Engineering University College London London WC1E 6BT UK; ^2^ Advanced Propulsion Lab, Marshgate University College London London E20 2AE UK; ^3^ The Faraday Institution, Quad One Harwell Science and Innovation Campus Didcot Oxon OX11 0RA UK; ^4^ Diamond Light Source Harwell Science and Innovation Campus Didcot Oxon OX11 0DE UK; ^5^ Department of Engineering Science University of Oxford Oxford OX1 3PJ UK; ^6^ The ZERO Institute University of Oxford Oxford OX2 0ES UK

**Keywords:** batteries, degradation, image analysis, micro‐CT, NMC

## Abstract

During battery operation, cracking occurs in Nickel Manganese Cobalt (NMC) oxide secondary particles. Cracked particles appear darker in micro‐computed tomography (micro‐CT) images due to the partial volume effect, where voxels containing both void and solid yield intermediate grey‐levels. This work presents an automated method for tracking grey‐level changes caused by this effect in large, statistically meaningful micro‐CT datasets containing over 10 000 individual particles. It extends earlier work using the GREAT algorithm to analyze NMC particles in tomography images. The new GREAT2 algorithm increases processing speed, from around 1,400 particles per day with GREAT to over 10 000 particles in under a minute. Furthermore, this work introduces methods for automated tracking of grey‐level intensity changes in individual particles through different states of charge in an operando experiment. This capability enables temporal analysis of particle degradation mechanisms. Additional data processing methods are presented that extract useful insights. Through this work we show that the large sample sizes, enabled by this method and GREAT2, allow for statistically robust analysis of particle populations. These advances significantly accelerate the tomographic study of cracking in battery electrodes. The GREAT2 algorithm and associated workflows have been made available as the GRAPES Python toolkit.

## Graphical abstract

This paper presents a new approach to quantifying damage in Nickel Manganese Cobalt (NMC) battery particles using grey‐level analysis of micro‐CT images. By leveraging the partial volume effect, the authors track subtle changes in particle degradation, even in sub‐resolution cracks. The newly developed GRAPES toolkit processes large datasets quickly, allowing high‐throughput analysis of over 10 000 particles. This method offers valuable insights for battery research, optimizing materials, and enhancing predictive models.

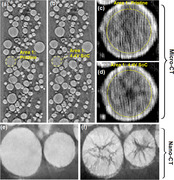

## 1 Introduction

The development of battery electric vehicles is critical in efforts to decarbonise transport^[^ ^1^ ^]^ and lithium‐ion (Li‐ion) batteries have become synonymous with this effort. As a result, much research has focused on developing high‐rate and capacity cathode materials for Li‐ion batteries.^[^ ^2^ ^]^ The high nickel content in NMC811 (Li_*x*_Ni_0.8_Mn_0.1_Co_0.1_O_2_) cathodes provides good performance by these metrics^[^ ^3^, ^4^ ^]^ and the reduction in cobalt compared to other NMC family materials offers important supply chain benefits.^[^ ^3^, ^5^ ^]^ However, these materials suffer from a wide range of degradation mechanisms that lead to capacity fade after many charge/discharge cycles. For example, undesirable phase transitions,^[^ ^6^, ^7^, ^8^ ^]^ increased transition metal dissolution,^[^ ^3^, ^9^, ^10^ ^]^ gas release,^[^ ^11^, ^12^ ^]^ and the formation of cracks,^[^ ^13^, ^14^, ^15^, ^16^ ^]^ have all been shown to lead to capacity fade, especially in nickel rich chemistries. Cracking in particular has been shown to be one of the primary considerations in capacity fade.^[^ ^17^ ^]^

**Figure 1:**
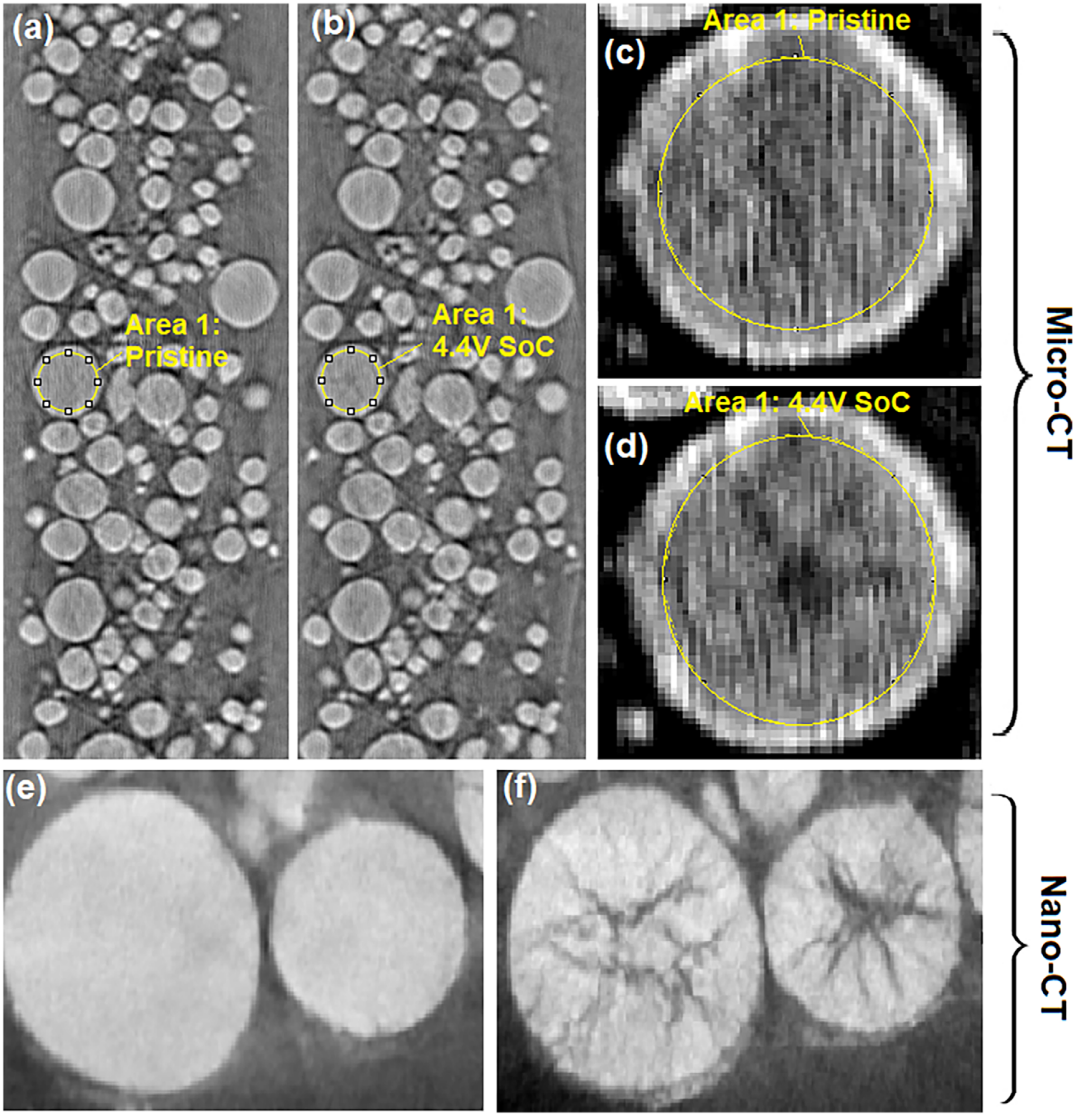
The partial volume effect of sub‐resolution cracks in Micro‐CT images of NMC particles. a) Micro‐CT slice showing pristine NMC particles with Area 1 highlighted, b) the same micro‐CT ROI at 4.4V SoC with Area 1 highlighted. c) A high contrast & magnified micro‐CT view of area 1 when pristine. The mean grey‐level in area 1 is 148.0. d) A high contrast & magnified micro‐CT view of area 1 at 4.4V SoC. The mean grey‐level in area 1 is now 147.1. e) Nano‐CT of a pristine NMC particle, f) Nano‐CT of a NMC particle at 4.5V SoC. Nano‐CT courtesy of Parks et al.,^[^ ^18^ ^]^ data available at ref. [19].

X‐ray Computed Tomography (XCT) has been used extensively to study NMC secondary particles.^[^ ^20^ ^]^ When studying the cracks that form at the particle scale during cycling, nano‐resolution XCT (nano‐CT) can be utilized.^[^ ^18^, ^21^, ^22^ ^]^ In previous work from our group, Parks et al.^[^ ^18^ ^]^ showed that electrochemical cracking occurs at high State‐of‐Charge (SoC), above c‐lattice collapse,^[^ ^23^ ^]^ even during the first charge/discharge cycle. This work showed that cracking was most intense at the center of NMC811 particles with smaller cracks advancing radially outward from the center. This demonstrated the capability of nano‐CT to directly observe different cracking mechanisms in NMC secondary particles. This is shown in **Figure** **1**e,f where we have reproduced nano‐CT slices of pristine and 4.5V SoC NMC particles from Parks et al.

However, whilst nano‐CT is an important tool for studying cracking mechanisms on the particle level, it has limitations at the electrode or cell level. Principally, nano‐CT has a significantly smaller field‐of‐view (FOV) compared to micro‐CT, which means fewer particles can be analyzed. As a result, drawing statistically significant conclusions about larger, real, populations of particles, and variations within these populations, is challenging. Furthermore, due to the much larger *Surface Area: Volume* ratio of nano‐CT samples, a larger fraction of particles may be damaged during sample fabrication. The smaller FOV also introduces challenges to in‐situ and operando cell design, meaning that quasi in situ methods such as that developed by Parks et al.^[^ ^18^ ^]^ are often used in place of in situ cells. Finally, it should be noted that nano‐CT microscopes are in general more expensive, less available, and have longer scan times compared to micro‐CT microscopes. As a result it can be difficult to get enough microscope time to image many samples without applying for synchrotron beamtimes, which have similar availability issues.

Micro‐CT, on the other hand, has a larger FOV and thus has excellent sample level statistics. However, it is limited by its resolution when studying cracking on the particle level. As a result, it is difficult to resolve cracks sufficiently so that they can be reliably segmented. Many researchers have segmented resolvable cracks in micro‐CT datasets and have used advanced methods to help detect and segment these cracks.^[^ ^24^, ^25^, ^26^, ^27^, ^28^ ^]^ However, these studies have been constrained to analysis of large cracks that may not be representative of the whole sample. We may reasonably expect to miss cracks that are representative of lower or intermediate damage with such methods.

In this work, we demonstrate methods for quantifying the degradation of NMC particles, at both particle and sample levels, and at low and intermediate damage states. This was achieved in large micro‐CT datasets of 10 000+ particle instances. The method assumes secondary particles with cracks will have observably lower grey‐levels with absorption contrast CT compared to an otherwise identical pristine particle, see Figure 1. This is due to the ‘partial volume effect’ where voxels representative of space with both void and solid phases result in grey‐levels that are intermediate between voxels representative of void or solid only, as previously described in Wade et al.^[^ ^29^ ^]^ We show that although this grey‐level change is small on the particle level (when averaged over a whole particle), when considered over a sample of thousands of particles, it is statistically significant. Lithiation and de‐lithiation also effect the attenuation cross‐section of the material, however, for relatively heavy materials, like NMC, at beam energies typical for micro‐CT, this effect is negligible. We demonstrate this with an approximation of the attenuation cross sections of lithiated and de‐lithiated NMC811 in appendix B. Hence, by tracking grey‐level change we have a proxy for tracking cracking in particles.

In this work, we achieved this using our newly developed GRAPES (**GR**ay‐level **A**nalysis of **P**articl**ES**) python toolkit that rapidly transforms large tomography datasets into easily queryable tables for tracking grey‐level change. As part of this work, we improved the GREAT algorithm previously demonstrated by our group in Wade et al.^[^ ^29^ ^]^ Our new GREAT2 algorithm, used in the GRAPES toolkit and presented here, is able to process large datasets rapidly. Wade et al. reports processing about 1400 particles per day with GREAT. By comparison the updated GREAT2 algorithm is able to process ≈10 000 similar particles in 37 s. The GRAPES toolkit calculates a variety of useful particle characteristics and contains utility functions designed to help users process the dataset created. As we show in this article the GRAPES toolkit can work effectively with operando data where the aim is to track grey‐level change in the same particle at different time steps. Alongside the manuscript, the GRAPES python toolkit and a user friendly GRAPES graphical user interface (GUI) have been made publicly available.

Here, we focus on the data analysis method developed to study grey‐level change in XCT images of particles due to degradation, and prove the statistical significance of this approach in the case of NMC811 secondary particle cracking. Presented here are two case studies used to demonstrate this method. Case study 1 looks at the degradation of an NMC811 cathode that was harvested from a commercial cell (LiFun Technologies, China). The sample was imaged ex situ in both the pristine state and at the top of charge. The tomography was performed using a commercially available laboratory micro‐CT microscope. Case study 2 looks at crack formation during the first charge/discharge cycle in NMC811 particles. The data set is operando and looks at a single region of interest (ROI) that degrades over time at multiple different SoC. In this case study, we demonstrate how 10 000+ particles can be automatically tracked through these SoC. In this case, the tomography was performed at the Diamond Light Source (DLS) synchrotron I13‐2 beamline.

## 2 Experimental Section

Please see Appendix A for details of cell fabrication and cell electrochemical history.

### 2.1 Micro‐CT Acquisition

In case study 1, ex situ micro‐CT was acquired using a ZEISS Xradia 620 Versa at the UCL Center for Correlative X‐ray Microscopy. The samples consisted of an NMC811 cathode that had been harvested from a commercial cell (LiFun Technologies, China). An 80 × 250 µm protrusion, or tab, was laser cut at the top of the electrode. This tab geometry was ideal for tomographic imaging that had been used in previous studies.^[^ ^30^, ^31^ ^]^ This laser cut, pristine, ex situ electrode was then mounted in the microscope and the tab was centered in the FOV for imaging. A total of 1601 projections were acquired over a 360 degree angular range and each projection had an exposure time of 45 s. An 80 kV accelerating voltage was used to create an X‐ray beam that illuminated the sample at a source‐to‐sample distance of 30.49 mm. The 40 × objective lens coupled with the 2048 px CCD detector was used at a sample‐to‐detector distance of 16.48 mm, this resulted in a pixel size of 220.3 nm. Once the tomogram was acquired, this electrode was removed and assembled into a single‐layer pouch cell. The cell was then charged to 4.5 V at a rate of C/50. The pouch cell was then disassembled, the electrode removed, and then re‐imaged using the same acquisition parameters as described above for the pristine state.

**Figure 2:**
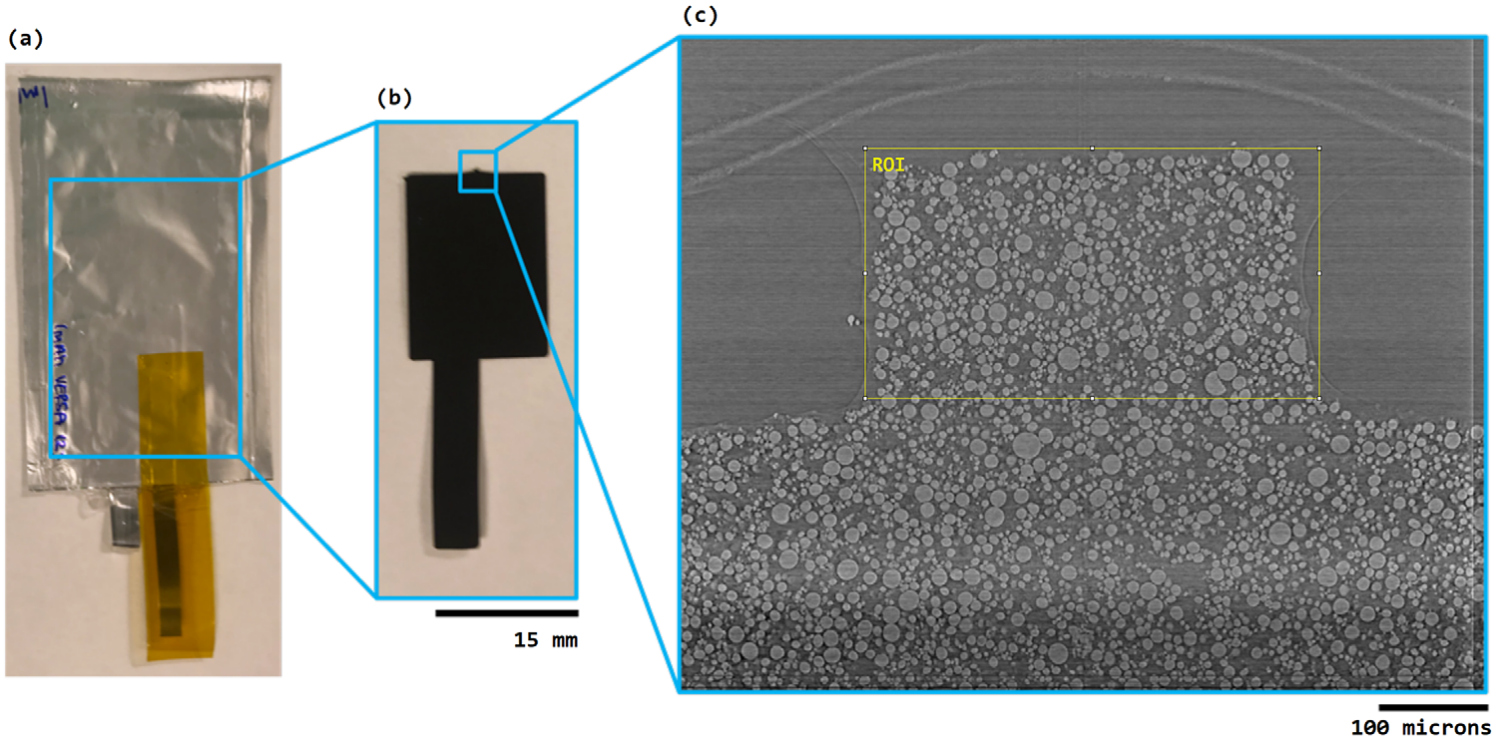
Case Study 2: The fabrication of an in‐situ imaging pouch cell. a) An operational single layer pouch cell, b) the electrode that is inserted in the pouch cell, the laser cut imaging tab is visible at the top of the image, c) reconstructed slice from a tomogram acquired during the experiment showing the imaging tab. This imaging tab is our region‐of‐interest (ROI).

In case study 2, operando micro‐CT of NMC811 at multiple SOC was acquired at the DLS i13‐2 beamline.^[^ ^32^ ^]^ A bespoke pouch cell sample holder made of Polyether Ether Ketone (PEEK) with an aluminium x‐ray window compressed the pouch cell throughout the experiment. This allowed the same ROI to be scanned repeatedly without removing the cell between scans. The ROI was a tab that had been laser cut from the electrode so that it extended above the rest of the pouch cell, which is shown in **Figure** **2**. Electrical connections allowed for electrochemical control of the cell for charge/discharge between specific voltages (vs. Li/Li+). The ROI was first scanned in the pristine state, and then after being charged and held at; (charging) 4.0, 4.1, 4.2, 4.3, 4.4 V, and (discharging) 4.3, 4.2, 4.1, 4.0, 3.8, and 2.5 V. However, in this paper, the data analyzed was limited to tomograms acquired at pristine, 4.0, 4.4, and 2.5 V after discharge. Scanning was performed at open circuit voltage, after a voltage hold until current had dropped to 10% of the charging current. A pco.edge 5.5 scintillator‐coupled detector with a 10 × objective lens was used which resulted in an effective pixel size of 325 nm. The FoV was 0.83 × 0.70 mm. For each tomography scan, 3000 projections were acquired between 0–180° with 0.8 s exposure. The ‘pink beam’, which makes use of multiple harmonics from the undulator, was utilised, resulting in higher flux than the monochromatic beam. The beam had an average energy of ≈27 keV.

### 2.2 Image Processing

**Figure 3:**
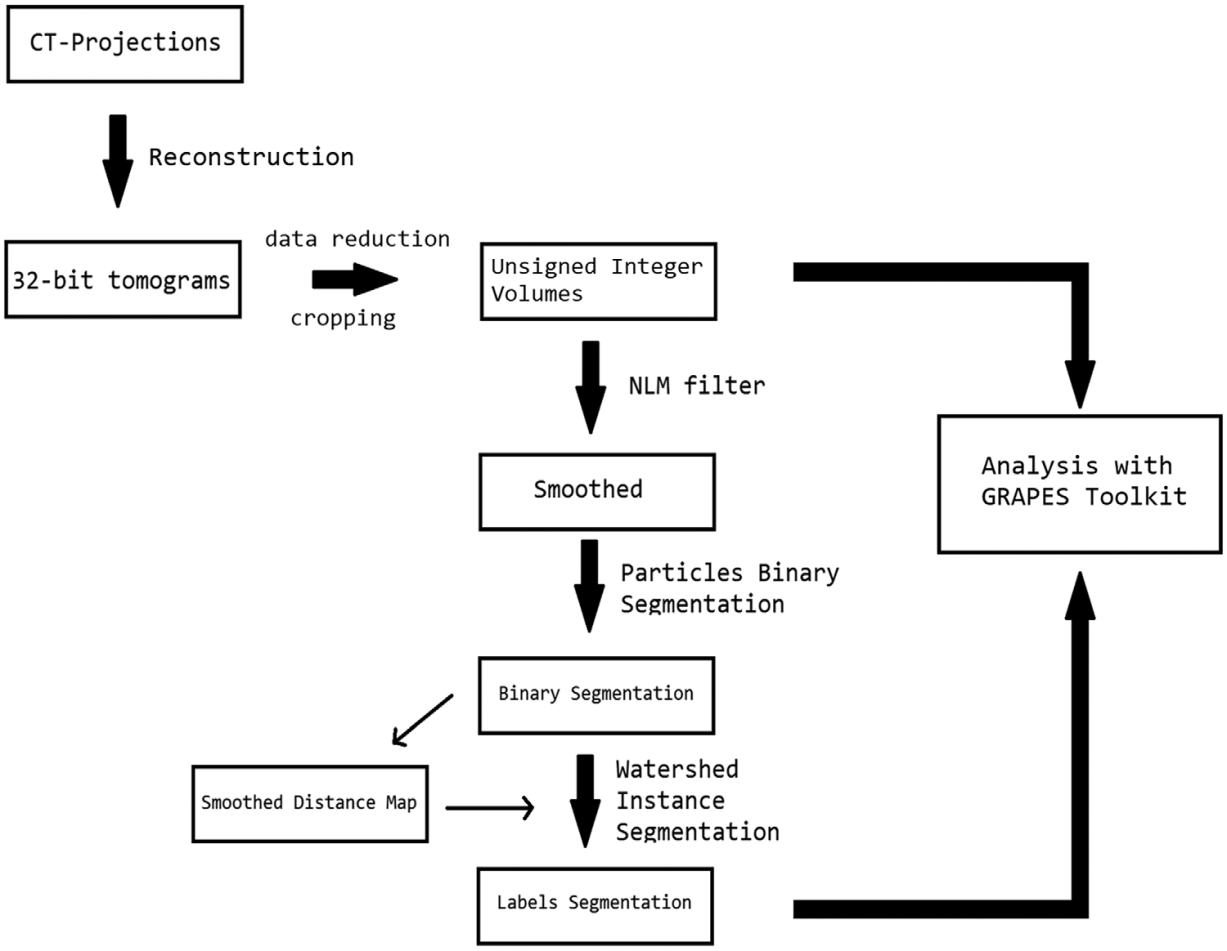
The general workflow for processing image data from both case studies. For both case studies it is critical to have a good particles labels segmentation where each particle is accurately separated and assigned a unique 16‐bit label. This segmentation and a correlated eight‐bit or 16‐bit (unsigned integer) unsmoothed grey‐level image are used as inputs into the later GRAPES analysis.

In case study 1, the general image processing workflow follows that shown schematically in Figure 3. Projections were reconstructed into image volumes using the filtered back projection algorithm and the data was cropped to the ROI. The CT volume was then segmented into a binary segmentation (of solid & void) using a threshold value. Morphological binary erosion and dilation were required to fill binary ‘holes’ and remove ‘islands’ from the binary segmentations. The binary segmentation was used to create a label segmentation where each particle was assigned its own 16‐bit unsigned integer label. This was achieved using the watershed threshold method from scikit‐image on a smoothed distance map.^[^ ^33^ ^]^ The unsmoothed grey‐level and label segmentation were then used as inputs to the GRAPES analysis. Figure 5 shows example segmentations.

**Figure 4:**
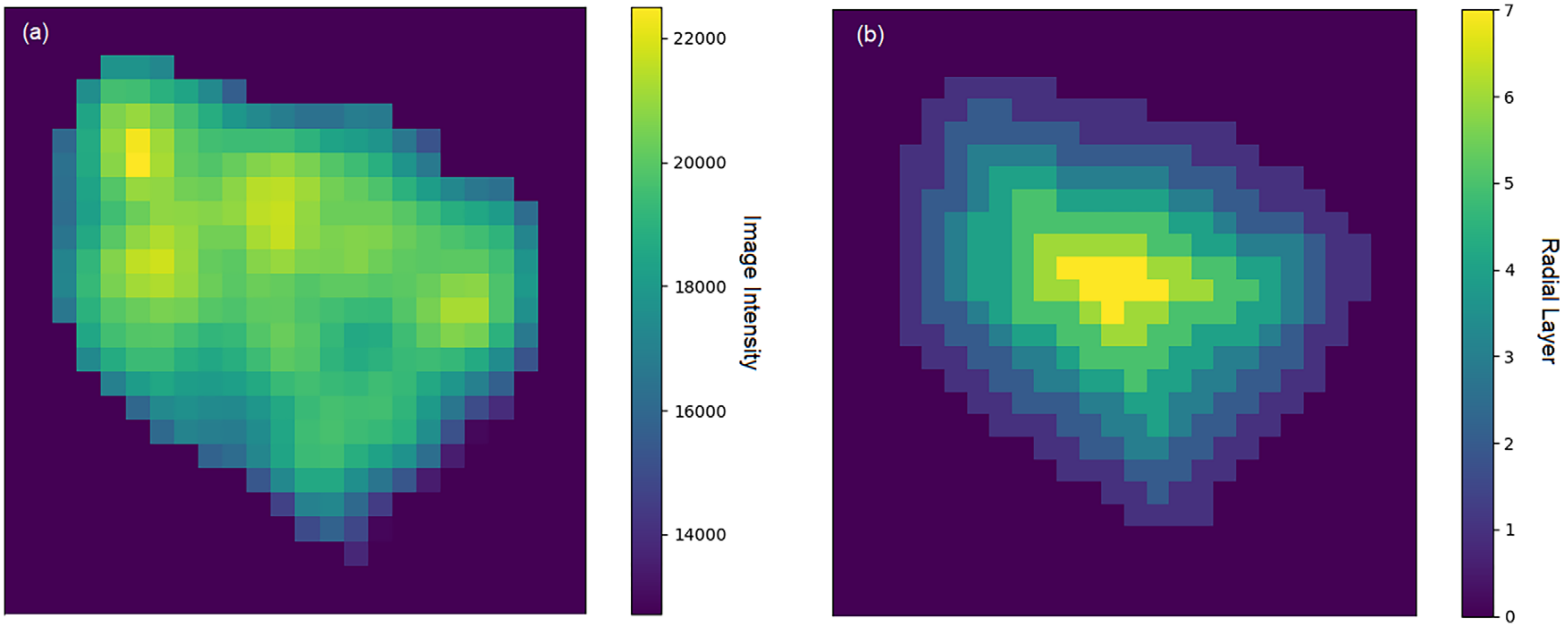
a) grey‐level image intensity of a particle b) radial layers of the particle as calculated by the toolkit. Both visualized using the viridis colormap.

**Figure 5:**
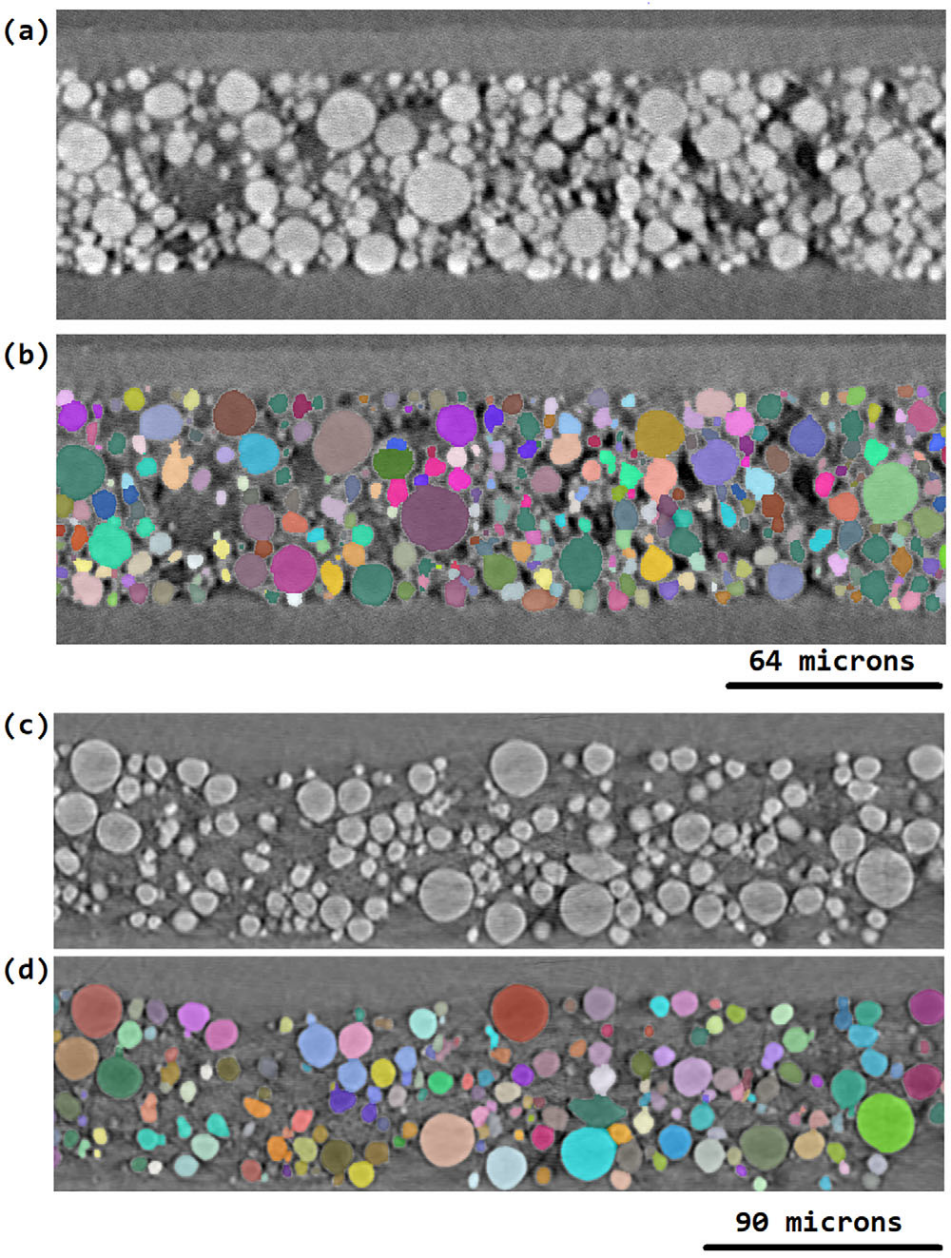
Case Study 1: a) grey‐level image of 4.5 V ex situ sample b) the corresponding label segmentation overlayed onto the grey‐level image. Case Study 2: c) unsmoothed grey‐level image from the in situ study at a 4.4 V SoC d) the corresponding labels segmentation overlayed onto the grey‐level image.

In case study 2, the image processing workflow also follows that shown schematically in Figure 3, but with an additional image registration step to align the data acquired at different SoC. Projections were processed and reconstructed using Savu data pipelines at the I13‐2 beamline.^[^ ^34^, ^35^ ^]^ Projections were processed with dark‐flat field correction and the ring removal algorithm from Vo et al.^[^ ^36^ ^]^ and were reconstructed with TomoPy's GridRec algorithm.^[^ ^37^ ^]^ The reconstruction was computed with limited angles (3–177°) because of projections at extreme angles being obscured by the sample holder. The reconstructed data was cropped to contain the ROI only. This data was registered such that corresponding pixels represented the same real space in tomograms taken at different SoC. This was achieved using the SimpleITK python library.^[^ ^38^ ^]^ This registered data was converted to eight‐bit unsigned integer volumes and smoothed using the fast non‐local means (NLM) algorithm from scikit‐image which was parallelised using the python concurrent.futures module.^[^ ^33^ ^]^ A threshold was used to calculate a binary segmentation of solid and void. Morphological binary erosion and dilation was required to fill binary ‘holes’ and remove ‘islands’ from the binary segmentations. When calculating the labels segmentation, it was critical to preserve particle labels between time steps so that they could be tracked at different SoC. The same watershed segmentation using a smoothed distance map (as in case study 1) was used to compute the label segmentation in the pristine state. However, for the remaining tomograms (acquired at different SoC), the label values were initiated using seeds labelled with the corresponding value from the pristine labels segmentation. This enforces the same labels across the tomograms but relied on good registration between the tomograms and relatively little particle drift during the experiment. The unsmoothed eight‐bit unsigned integer grey‐level data and the labels segmentation was then used in the GRAPES analysis (Figure 5 shows example segmentations).

### 2.3 The GRAPES Toolkit

The GRAPES toolkit was a python toolkit we developed to transform large datasets of separated particles into easily queryable tables of particles, particle characteristics, and radial particle characteristics. Particle characteristics relating to radial layers within particles were calculated using an updated version of the original MATLAB based GREAT algorithm, which was described by Wade et al.^[^ ^29^ ^]^ The new GREAT2 algorithm used as a parallelized multi‐label anisotropic 3D euclidean distance transform (MLAEDT‐3D)^[^ ^39^ ^]^ in‐place of binary erosion to accelerate computation. GREAT2 parses each particle label for which the Euclidean Distance Transform (EDT) was calculated and rounded to the nearest integer in order to calculate ‘layers’, see **Figure** **4**. Care was taken to develop an algorithm that used vectorized operations and avoided nested loops for rapid computation with the large 3D image datasets typical in tomographic imaging. Once the radial layers are extracted, the mean grey‐level and mean normalized grey‐level were calculated in each radial layer and store this in the table. In addition, the table was populated with the mask, grey‐level, and EDT image of each particle in a bounding box. Scikit‐image's regionprops function^[^ ^33^ ^]^ was now utilized in the toolkit to include a broader range of particle characteristics from grey‐level images of particles and their corresponding labels segmentation. These include characteristics such as volume, diameter, sphericity, surface area, centroid, local centroid, mean grey‐level intensity, grey‐level intensity standard deviation, and more. Beyond the calculation of particle characteristics tables, the toolkit now contained a number of utility functions that help subset, analyze, visualize, and explore both in situ and ex situ datasets of particles. When benchmarked, the computation of a GRAPES table took 36 s, with a peak memory usage of 1600 MiB, this for a dataset that contained ≈ 10 000 particle labels with an average pixel volume of 25,300 px. An Intel Xeon 2.70 GHz CPU processor on a workstation with 128 GB of installed RAM was used for this computation. All 24 cores were used in parallel during the parallel computation of the label EDTs. Alongside this article, this code is made available in the form of the GRAPES python toolkit and a GUI.

### 2.4 Data Cleaning

Tables of particles and particle characteristics were calculated from the unsmoothed grey‐level images and the corresponding label segmentations for both case studies by using the grapes toolkit, see **Figure** **5**. A critical step in processing data like this is to clean the dataset and remove edge cases. In case study 2 (the operando and time resolved case) one of the most critical steps was to make sure that particle labels were consistent between the tomograms that represented the ROI at different SoC. This was mostly achieved by the segmentation; however, segmentation errors still had to be removed from the analysis. This was achieved by removing any particle labels from the analysis that did not exist in all the tables representing different SoC (by dataframe merging). Furthermore, it was found that it was necessary to remove any particles that shared an edge or were close neighbors with a particle label that was not consistent across the dataset (i.e., one of the particles removed in the previous step). This was because particle labels surrounding these inconsistent labels often absorbed the pixels otherwise assigned to the inconsistent label. In addition to this, in both case examples, any particle labels from the analysis that shared an edge with the edge of the image ROI were removed. This was important as it removed partial particle volumes from the analysis. The data was then ready to be queried in order to extract insights on NMC secondary particle degradation.

## 3 Results

**Figure 6:**
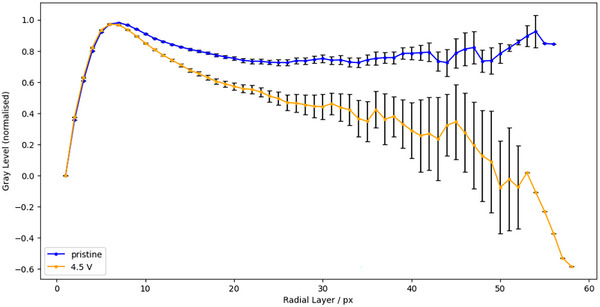
Case Study 1: The normalized mean grey‐levels in radial layers for the pristine and cycled data from case study 1. Radial layer zero refers to particle surfaces, with the following layers moving toward the particle cores. There are lower grey‐levels in the cycled data which is indicative of cracking and damage. Included are standard error bars (±2SE) for every radial layer.

In case study 1 we aim to show that this method can be used to detect damage due to cracking and void formation in NMC particles by comparing the pristine and 4.5 V SOC images. In this case the images were not registered, therefore particles grey‐level cannot be tracked in specific particles at different SoC. Therefore, we are interested in the detection of damaged regions within particles, rather than the distribution of grey‐levels between particles. This means we are interested in detecting regions within particles that have significantly lower grey‐level than the surrounding material. For example, the dark patch in the centre of Figure 1d. In order to do this, radial grey‐levels from each particle were extracted and normalised such that the highest radial layer grey‐level value is normalized to *one* and the radial grey‐level at the particle surface is normalized to 0. Across the sample set of particles, a mean normalized grey‐level for each radial layer was calculated. A plot of such normalized mean radial grey‐levels is shown in **Figure** **6**. In this example the set of particles was sub‐set to just include the largest quartile of particles, including 4532 pristine particles and 4375 charged particles. In Figure 6 there is a trend by which radial layers toward the center of the cycled particles have, on average, relatively lower grey‐levels compared to the same region in the pristine dataset. This is characteristic of damage in these materials, where we expect to see lower grey‐level cracks and voids appear in the 4.5 V SoC particles, with these emanating from the particle centers.^[^ ^18^ ^]^ Furthermore, the SE in radial layer grey‐levels in the 4.5 V SoC image is higher, particularly towards the center of the particles, this shows that average grey‐level in radial layers is quite inconsistent compared to the pristine state, suggesting a varying amount of damage between particles. This underlines the importance of using characterization techniques with good sampling statistics in terms of the number of particles under study.

**Figure 7:**
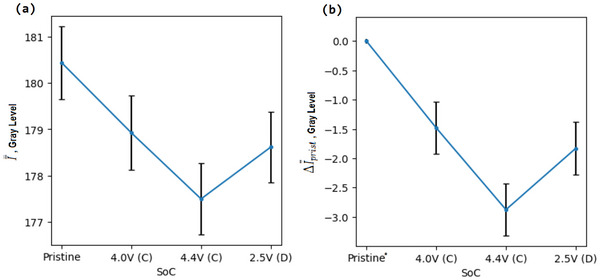
Case Study 2: a) A plot showing the $\bar{\bar{I}}$ at a range of SoC. Error bars plot ±3SE. b) A plot showing the $\Delta {\bar{\bar{I}}}_{prist}$ at a range of SoC. Error bars plot ±3SE. In both examples the smallest volume quartile of particles was excluded. Additionally, the outer five pixels in each particle were removed from the analysis (see Figure 8b for relevant ROI). *X*‐axis labels refer to the SoC, the letter in brackets refers to phase of the cycle; charging (C), discharging (D). * By definition the $\Delta {\bar{\bar{I}}}_{prist}$ at the the pristine state equals zero.

**Figure 8:**
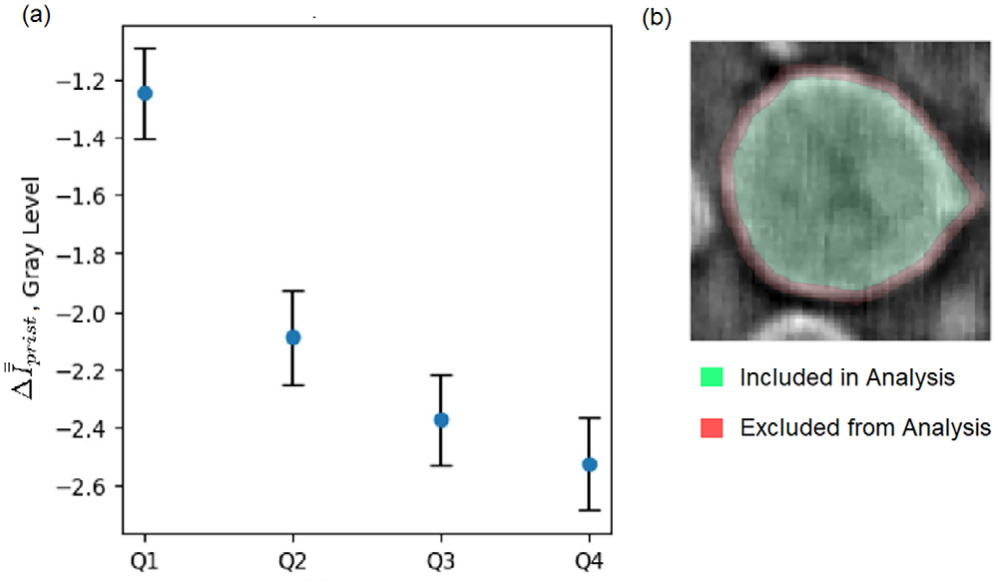
Case study 2: a) A plot showing the $\Delta {\bar{\bar{I}}}_{prist}$ inside x‐position quartiles (Q1, Q2, Q3, Q4). The error bars plot ±1SE. The smallest volume quartile of particles was excluded. Additionally, the pixels ⩽ 5 radial layers from the edge of the particle were not considered, b) Schematic showing how pixels in the first five radial layers of a particle are removed from analysis.

An operando method where the exact same set of particles can be analysed at each step is demonstrated for case study 2. In this case, by careful segmentation and the removal of inconsistent particle labels, it was possible to see how the grey‐level in an identical set of particles changed during charge/discharge over 1 cycle. **Figure** **7**a tracks how the ‘mean of mean particle grey‐levels’, or $\bar{\bar{I}}$, changes in this set of particles at different SoC. $\bar{\bar{I}}$ was calculated by calculating the mean grey‐level within a ROI for each particle (see **Figure** **8**b for ROI), and then calculating the mean of the population of particles at each SoC. In Figure 7 it is observed that there is a general trend where grey‐levels dropped during charging, and then recovered during discharging. This indicates that damage, such as cracking and void formation, occurs during charging and then ‘healing’ occurs during discharging. Healing in this case refers only to the spatial closing of cracks and voids and not the resintering of primary particle grains.

However, a more advanced analysis can be performed on a dataset that has particle labels matched across images. As a result of this, it was possible to track grey‐level change in specific particles at different SoC. For every particle in the dataset, the mean grey‐level change relative to its mean grey‐level in the pristine state was tracked, or $\Delta {\bar{I}}_{prist}$. (Again, when calculating the mean particle grey‐levels the ROI of each particle excluded pixels at the edge of the particles due to their phase contrast enhancement, see Figure 8b). Calculating the mean $\Delta {\bar{I}}_{prist}$ of a set of particles gives the ‘mean of mean particle grey‐level change versus pristine’ or $\Delta {\bar{\bar{I}}}_{prist}$. This is subtly different from simply tracking the $\bar{\bar{I}}$ as in Figure 7a and is generally more precise, as shown in Figure 7b, where the associated standard error is lower. This is because this method normalizes out the large variation in mean particle grey‐levels within the sample, thereby reducing sampling error. Tracking the $\Delta {\bar{\bar{I}}}_{prist}$ is particularly useful when comparing different subsets of data. This is because when doing so a low $\bar{\bar{I}}$ does not necessarily indicate that a drop in grey‐level has occurred in this subset as the initial state is unknown (i.e., the subset may have started with a low $\bar{\bar{I}}$ in the pristine state).

An example of the advantage of tracking $\Delta {\bar{\bar{I}}}_{prist}$ is shown in Figure 8. In order to determine the relationship between grey‐level change and the spatial location of particles within the electrode, four subsets of particles were compared. These subsets contained particles from different *x*‐position quartiles. The first quartile is furthest from the current collector and the fourth quartile is closest to the collector. Plotted in Figure 8 is the $\Delta {\bar{\bar{I}}}_{prist}$ for each quartile. It was observed that the smaller *x*‐positions values (further from the current collector) have a lesser drop in grey‐level compared to those next to the current collector. Implying that cracking reduces with distance from the current collector in this case study. It should be noted that in modelling literature it is predicted that particles furthest from the current collector would experience more damage when *C* rate was ⩾3*C*, whilst at 1*C* a homogenous distribution of cracking was predicted.^[^ ^40^ ^]^ Our findings above contradict this where we observed higher levels of cracking closer to the current collector, albeit at a lower *C* rate of *C*/3. We suggest that this may be due to particles further from the collector surface having poorer than expected electrical contact, which may result in partial charging and thus less observed damage. This effect may be exacerbated due to the sample being uncalendered, a choice made to avoid the introduction of mechanically‐induced cracks due to calendering, as we wanted to isolate electrochemical cracking in this experiment.^[^ ^41^ ^]^

**Figure 9:**
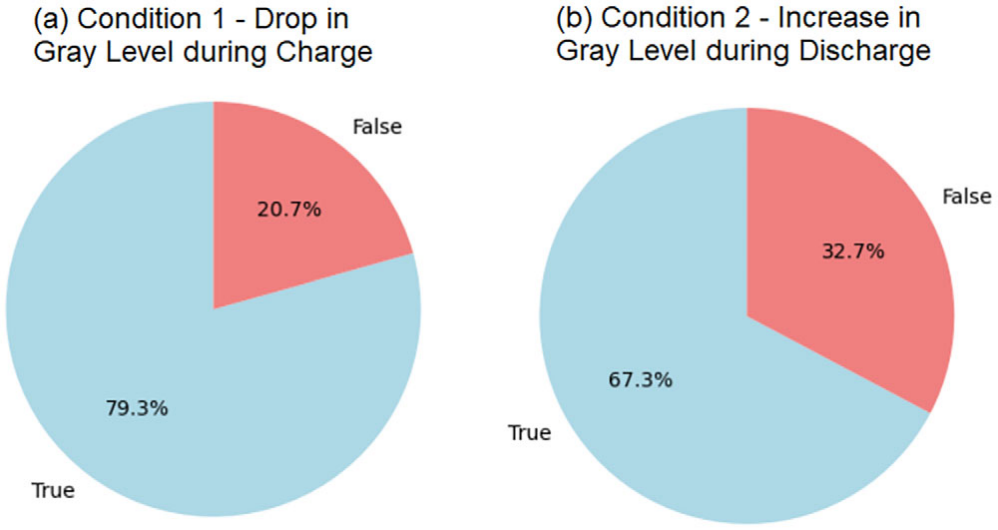
Case study 2: a) A pie chart showing the percentage of particles that have a lower grey‐level after charge at 4.4 V than in the pristine state. b) A pie chart showing the percentage of particles that have a higher grey‐level at 2.5 V after discharge than at 4.4 V.

Additionally, it is possible to use the mean particle grey‐level parameter to analyze the behavior of particles based on conditions. It is easy to calculate the percentage of particles that obey a particular condition, for example, ‘Does the particle have a higher mean grey‐level in the pristine state compared to the 4.4 V SoC’ (Condition 1) and ‘Does the particle have a lower mean grey‐level at the 4.4 V SoC compared to 2.5 V SoC after discharging’ (Condition 2). The percentages of the particles that obey the conditions are plotted as pie charts in **Figure** **9**. Again, the analysis here removed the smallest quartile of particles and did not consider particle edges because they mostly consisted of phase contrast enhanced pixels (see Figure 8). From this analysis in Figure 9a, it is observed that a significant majority of particles obey condition 1, suggesting that these particles are becoming damaged by cracks and voids that lower the particle density and, therefore, grey‐level. Figure 9b shows that a smaller majority of particles obey condition 2. This suggests that during discharge, the particles ‘heal’ as the cracks and voids in the particle move back together, likely due to the expansion of primary particle crystallites during delithiation.^[^ ^42^, ^43^ ^]^

**Figure 10:**
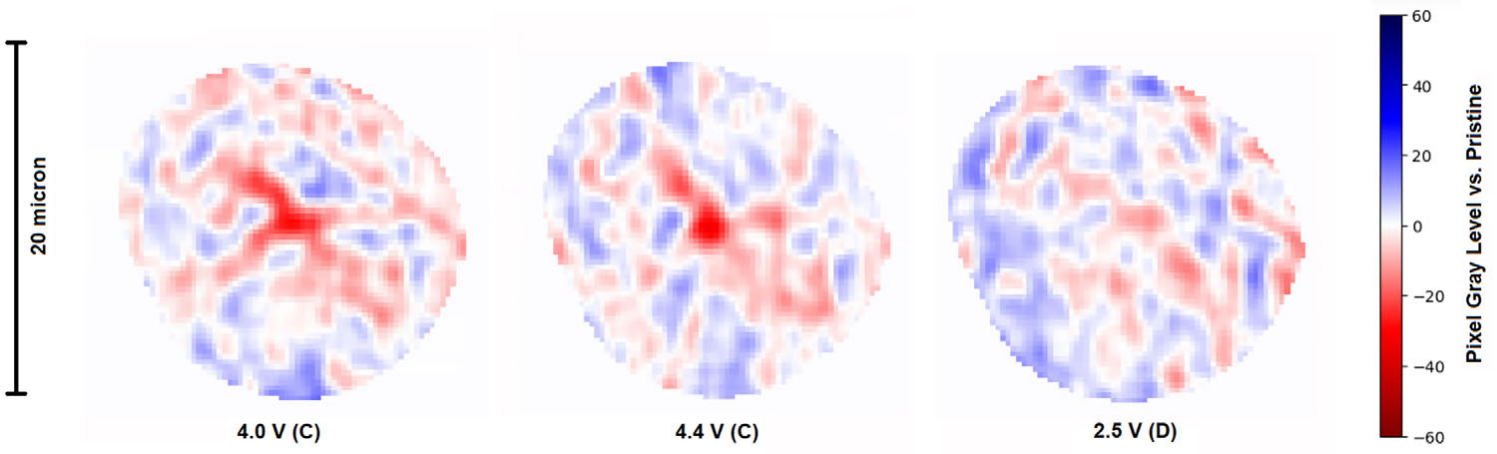
Case Study 2: Intra‐particle visualization of grey‐level change. The Figure shows the pixel level grey‐level change versus pristine for a particle at 4.0 & 4.4 V during charge and 2.5 V during discharge. Positive changes are plotted in blue, and negative changes are plotted in red. The outer five pixels of the particle are not plotted due to their high phase contrast enhancement.

Finally, it may seem naive to expect to be able to reliably detect particle‐level fluctuations of only a few eight‐bit grey‐level values when this is so close to the intensity resolution of an eight‐bit image. However, it is important to consider that the values being reported here are average values calculated over many pixels in a particle. Many fluctuations in grey‐level within the particle images are much larger, as shown in **Figure** **10**. In this figure, the pixel grey‐level change versus pristine for the same particle at different SoC is shown. The displayed images were calculated from first re‐registering the particle onto itself at the different SoC (even though the image was already registered, re‐registering just the particle bounding volume can be more accurate), then smoothing the images with a Gaussian filter, and finally subtracting the intersecting volumes of the pristine image from the SoC image. From this visualization of intra‐particle damage it was observed that damage primarily occurred in the center of the particle and that this damage was locally characterised by relatively large decreases in grey‐level (≈ 40 grey‐levels), thus demonstrating the ability of this technique to resolve intra‐particle damage and showing that the technique is not limited by the intensity resolution of an eight‐bit image.

### 3.1 Validation with Nano‐CT

**Figure 11:**
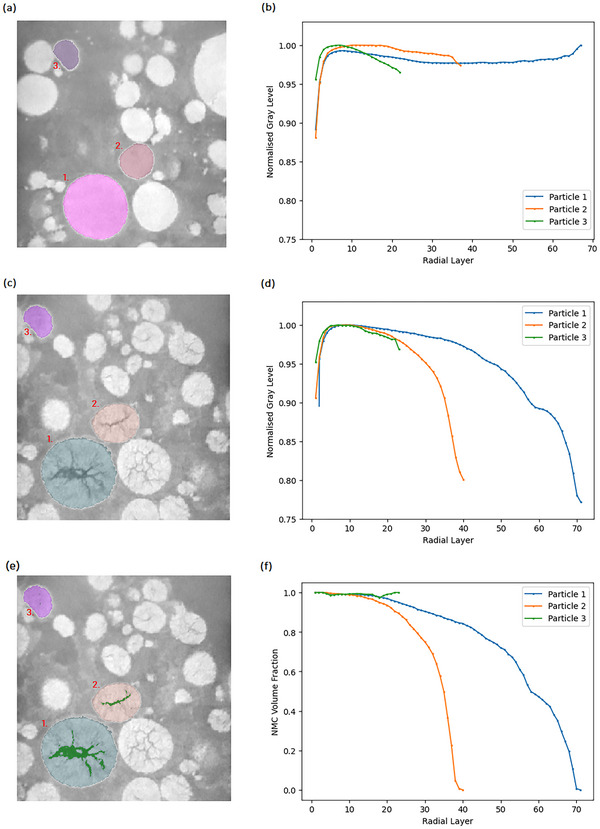
a) a nano‐CT slice with the label segmentation of three pristine NMC811 particles overlayed b) normalized mean gray level in the radial layers of the pristine particles. c) nano‐CT slice with the label segmentation of the same three particles after being charged to 4.4V. d) normalized mean gray level in the radial layers of the charged particles. e) the same nano‐CT slice of the charged particles with the label segmentation and crack segmentation (in green) overlayed f) volume fraction of NMC according to the crack segmentation in radial layers of the charged particles.

In order to further illustrate that the grey‐level intensity method detects cracking and void formation, and not other phenomena that may effect grey‐level intensity, such as as lithiation and delithiation, we validate our method with higher resolution nano‐CT imaging. This is shown **Figure** **11** where we demonstrate that the grey‐level method, see Figure 11c,d, is highly comparable to a method where the level of cracking is determined by segmenting the cracks in each particle, see Figure 11e,f. In this nano‐CT example the cracks are well resolved and segmentable, however, in the micro‐CT datasets discussed above, the grey‐level approach is more robust for less well resolved cracks that are challenging or impossible to segment. Additionally, we observe the same trend in this nano‐CT data to the micro‐CT data presented in Figure 6, where the normalized mean grey‐level in radial layers was averaged across a dataset of thousands of particles imaged with micro‐CT. In both, we observe that cracking occurs most intensely in the core of NMC particles and radiates outwards. The nano‐CT data used for this validation is courtesy of Parks et al.,^[^ ^18^ ^]^ data available at Ref. [19].

### 3.2 Measurement and Sampling Uncertainty

Understanding uncertainty is critical when analysing this data. Error bars are included in the figures (e.g., Figures 7, 8, and 8) to visually represent uncertainty. For example, in Figure 8, ±3 standard errors (SE) are plotted, indicating a 99.7% confidence interval for the population mean. Uncertainty in this study is categorized into two main types: measurement uncertainty and sampling uncertainty. These are combined to calculate the total uncertainty (*SE*_*tot*_) using the specific equations described below.

**Figure 12:**
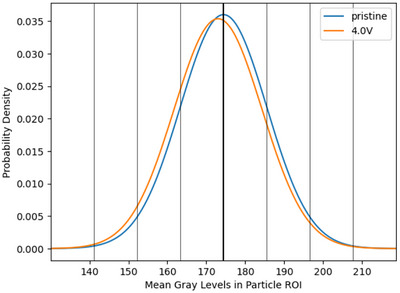
Case Study 2: The modelled normal distributions of mean grey‐level values in particle ROIs in the pristine and 4.0 V SoC samples. The vertical lines represent standard deviations from the pristine mean.

Measurement uncertainty refers to errors inherent in the imaging process. The standard error of the mean particle grey‐level (*SEM*_*P*_) for a single particle is calculated by dividing the noise level (σ_*bg*_), estimated from the standard deviation of the image background, by the square root of the number of pixels (*n*_pixels_) in the region of interest (ROI) for the particle:

(1) $$SEM_{P}=\frac{\sigma_{bg}}{\sqrt{n_{\text{pixels}}}}$$

To calculate the uncertainty in the change in grey‐level relative to the pristine state (*SEM*_Δ*P*_), the measurement uncertainties in the pristine state ($SEM_{P_{0}}$) and at a given SoC ($SEM_{P_{SoC}}$) are combined as follows:

(2) $$SEM_{\Delta P}=\sqrt{SEM_{P_{0}}^{2}+SEM_{P_{SoC}}^{2}}$$

The standard error of measurement of the $\bar{\bar{I}}$ and $\Delta {\bar{\bar{I}}}_{prist}$ parameters were calculated by combining the standard error values for each particle in the sample as in the equation below;

(3) $$SEM=\sqrt{\frac{1}{n}\sum_{i=1}^{n}{\left(SEM_{i}\right)}^{2}}$$

where *SEM* is the standard error of measurement of $\bar{\bar{I}}$ or $\Delta {\bar{\bar{I}}}_{prist}$, *n* is the number particles in the sample, and *SEM*_*i*_ is the *i* ^*th*^ *SEM*_*P*_ or *SEM*_Δ*P*_ value in our sample of particles (depending on the parameter being calculated).

Sampling uncertainty arises from variability in the dataset due to measuring only a subset of the total population. It is determined by dividing the standard deviation of the sample (σ) by the square root of the sample size (*n*):

(4) $$SES=\frac{\sigma }{\sqrt{n}}$$

Finally, the total uncertainty (*SE*_*tot*_) is calculated by combining the measurement uncertainty and the sampling uncertainty. This is expressed mathematically as:

(5) $$SE_{tot}=\sqrt{SES^{2}+SEM^{2}}$$

To illustrate the significance of observed grey‐level changes, a Z‐test was performed using data from Case Study 2. The null hypothesis (*H*_0_) assumed no change in the population mean grey‐level between the pristine state and 4.0 V SoC (i.e., any observed change is a random fluke of sampling), while the alternative hypothesis (*H*_*A*_) posited a decrease in mean grey‐level at 4.0 V. The Z‐statistic for this hypothesis test was calculated as:

(6) $$Z=\frac{{\bar{\bar{I}}}_{4.0V}-{\bar{\bar{I}}}_{\text{prist}}}{\sqrt{\frac{\sigma_{4.0V}^{2}}{n_{4.0V}}+\frac{\sigma_{\text{prist}}^{2}}{n_{\text{prist}}}}}$$

Substituting the observed values (${\bar{\bar{I}}}_{4.0V}=173.00$, ${\bar{\bar{I}}}_{\text{prist}}=174.41$, σ_4.0*V*_ = 11.27, σ_prist_ = 11.08, and *n*_4.0*V*_ = *n*_prist_ = 3650) into the formula produced a Z‐statistic of −5.40. The critical value for a one‐tailed test with a 5σ significance level is *V*_crit_ = −5.01. Since the calculated Z‐statistic is less than the critical value, the null hypothesis is rejected. This confirms that the observed decrease in grey‐level is statistically significant and not due to random sampling. Figure 12 illustrates this drop in population grey‐level. These analyses demonstrate that even small changes in grey‐levels, though subtle at the individual particle level, are statistically significant when evaluated across the large sample sizes enabled by this method. By carefully combining precise measurements with robust statistical analysis, the method ensures high confidence in detecting subtle changes in particle degradation.

### 3.3 Experimental Error

Several sources of experimental error were identified for this method, including error due to imaging artefacts, segmentation inaccuracies, inconsistent image acquisition, and user mistakes during data processing. Careful attention was paid to minimize these potential sources of error to ensure the reliability of the results.

Imaging artefacts such as rings, streaks, and double edges can impact the accuracy of grey‐level measurements. For instance, in Case Study 2, the use of limited‐angle tomography due to the experimental geometry introduced streaking artefacts. These were minimized by optimizing the experimental setup, including a beamline configuration that reduced the number of obstructed angles. When artefacts are unavoidable, advanced reconstruction techniques such as iterative algorithms and ring artefact removal could be employed to improve image quality.

Accurate segmentation of individual particles is critical for the analysis. In Case Study 1, segmentation errors were less problematic because particle labels were not required to match between pristine and cycled states. However, in Case Study 2, maintaining consistent particle labels across different SoC was essential. This was addressed using a seed‐based segmentation approach, and by removing inconsistent labels from the dataset with dataframe merging. The segmentation of smaller particles posed particular challenges due to phase contrast enhancement, and merged or erroneous labels. To mitigate this, the smallest quartile of particles was excluded from most quantitative analysis.

Consistency in imaging parameters was vital to ensure that observed grey‐level changes reflected true material change rather than variations in acquisition conditions. In Case Study 2, all images were acquired during a single beamline session, ensuring consistent beam energy, filtering, and sample‐to‐detector distance. This consistency was verified by observing stable histogram peaks in empty regions above the sample across scans. In Case Study 1, additional care was required due to potential variability in the lab‐based source. Flat‐field images and empty slices were examined for beam variation, which was found to be negligible.

To address potential errors during image processing, all image processing steps were automated using Python scripts to ensure repeatability. Visual validation of particle labels and segmentation results confirmed their consistency. For both case studies, datasets were cleaned by removing edge particles and inconsistent labels, ensuring that only reliable data were included in the analysis. Furthermore, care was taken during data comparison to normalize grey‐levels and reduce the impact of sample‐to‐sample variability. These measures collectively enhanced the robustness of the analysis and minimized the impact of experimental errors on the results. We believe these to be best practices when applying this method to similar data.

## 4 Conclusion

In this work, we have presented methods for quantifying damage due to cracking and void formation in NMC particles using grey‐level analysis of micro‐CT images. This study builds upon previous work by Wade et al.^[^ ^29^ ^]^ by introducing significant improvements in computation speed, handling operando datasets, and enabling automated tracking of grey‐level changes across a much larger number of particle instances (approximately 100,000). These advancements were achieved through the development of the GRAPES python toolkit, which includes the updated GREAT2 algorithm for rapid analysis of large tomography datasets. A GUI is also made available.

Two case studies were conducted to validate this approach. The first case study used ex situ data collected with a lab‐based micro‐CT system, while the second case study employed operando data acquired at a synchrotron beamline. Both studies revealed statistically significant grey‐level changes in NMC particles subjected to different states of charge. These changes were attributed to cracking and void formation, even when the cracks and voids were below the resolution of micro‐CT imaging. By leveraging the partial volume effect, the method demonstrated the ability to detect these sub‐resolution features, highlighting the power of micro‐CT's excellent sampling statistics to assess large populations of particles.

A key strength of this method is its ability to detect small, statistically significant changes in particle properties, even when individual variations are subtle. By analyzing thousands of particles within a single dataset, this method provides robust sample‐level insights into material degradation. For example, the operando study demonstrated spatially resolved damage patterns within the electrode, revealing that particles further from the current collector sustained less damage due to poorer electrical contact. These findings underscore the utility of this approach for linking particle‐level damage to cell‐level behavior.

This method has broader implications for battery research and development. By enabling high‐throughput analysis of particle degradation, it provides a valuable tool for assessing prototype materials, optimizing manufacturing processes, and developing predictive models of battery performance. Furthermore, the GRAPES toolkit simplifies data processing, making it accessible to a wide range of researchers and facilitating integration with machine learning models for advanced analysis.

Beyond micro‐CT, the methods and tools presented in this work have the potential to be valuable in other imaging and microscopy contexts where tracking grey‐level change in labelled regions is desirable. The methods and GRAPES python toolkit presented here are compatible with 2D images as well as images with anisotropic pixels/voxels. Thus, GRAPES is compatible with other sources of image data used to study electrochemically active particles, such as optical scattering microscopy^[^ ^44^ ^]^ and serial sectioning scanning electron microscopy.^[^ ^45^, ^46^ ^]^ Furthermore, we believe this method could be useful for analyzing data from more advanced synchrotron imaging techniques such as X‐ray Absorption Near Edge Spectroscopy (XANES) imaging and K‐edge subtraction imaging, where comparing grey‐level change between datasets acquired with different monochromatic beam energies implies chemical information about the sample.^[^ ^47^, ^48^, ^49^ ^]^ We anticipate that the GRAPES toolkit could be used in other fields of research. For instance, the ability to process large datasets of particles makes it suitable for fields such as catalysis, where micro‐CT and advanced imaging techniques are used to study the evolving structure and chemistry of active particles.^[^ ^50^, ^51^ ^]^ These capabilities position the GRAPES toolkit and the underlying analytical frameworks demonstrated here as useful tools for tackling challenges in imaging and microscopy across scientific disciplines.

## Conflict of Interest

The authors declare no conflict of interest.

## Author Contributions

M.P.J. developed the GRAPES software, GRAPES GUI and GREAT2 algorithm. M.P.J. carried out the data analysis. H.C.W.P. fabricated electrochemical cells. H.C.W.P. performed laboratory tomography. H.C.W.P., A.V.L., H.T.R., & C.T. performed synchrotron tomography. A.W. & T.M.M.H. developed the original GREAT algorithm from which the GREAT2 algorithm took inspiration. F.I. supervised laboratory tomography acquisition and provided insights into data interpretation. S.M. was beamline scientist who supervised and assisted with synchrotron tomography acquisition. P.R.S. & R.J. supervised throughout. Initial manuscript written by M.P.J., H.C.W.P. & R.J. All authors contributed to manuscript editing.

## Figures and Tables

**Figure A1:**
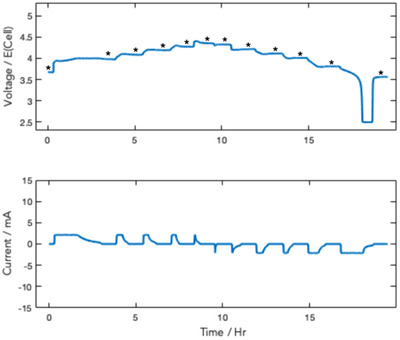
Case Study 2: Cell SoC history of the cycled sample. * indicates times where CT was acquired.

## Data Availability

The data that support the findings of this study are openly available in Micro‐Tomography of NMC811 Particles at https://doi.org/10.5522/04/25102538.v1, Ref. [52].

## Appendix A Cell Fabrication and Electrochemical History

**Case Study 1**: An electrode was sourced from a 220 mAh multi‐layered pouch cell (LiFun Technologies, China). One side of the electrode was cleaned of all active material using an appropriate solvent. This single‐sided electrode had an areal capacity of approximately 3.3 mAh cm^−2^. The electrode sheets were mounted on a translation table of a laser micro‐machining instrument (A Series, Oxford Lasers) equipped with a 532 nm laser and a spot size of 40 µm. The laser cut a series of lines, resulting in an electrode with a 500 µm × 250 µm tab protrusion at the top for imaging.

Following initial imaging, the electrodes were dried in a glass vacuum oven (Buchi, Germany) at 100°C overnight, then transferred to an argon‐filled glove box and assembled into a single‐layered pouch cell approximately 4 cm wide and 8 cm high. The cell was assembled with a lithium chip (Goodfellows, UK), using an excess of LiPF_6_ 1 M electrolyte in EC/EMC 3:7, with 2% wt vinylene carbonate (VC) (Soulbrain MI, USA). A tri‐polymer separator (Celgard R 2320, USA) electrically isolated the electrodes. After charging, the cells were disassembled in the argon‐filled glove box and left to dry before being placed into the custom‐made electrode holder. The electrodes were stored in the glove box until ready for imaging to avoid atmospheric damage.

The pouch cell was charged using a standard constant‐current‐constant‐voltage (CCCV) protocol on a Biologic BCS‐805 cell cycler at room temperature to 4.5 V (vs. Li/Li^+^) at a C‐rate of C/50, based on a calculated theoretical capacity of 9.9 mAh. Constant voltage was applied until the current dropped to C/100, after which the cell was disassembled as previously described.

**Case Study 2**: The positive lithium‐ion electrode samples, composed of lithium nickel manganese cobalt oxide (NMC811, NEI Corp.) and with an areal capacity of 2.2 mAh cm^−2^, were affixed to a petri dish using Kapton tape. The petri dish, along with the samples, was then securely attached to the translation table of a laser micro machining instrument (A Series, Oxford Lasers Ltd.). This instrument featured a 532 nm laser with a spot size of approximately 40 µm. The laser was programmed to mill a series of lines at a speed of 2 mm s^−1^ s. Subsequently, the resulting electrode was cut to dimensions of 15 × 20 mm (width x height), with a protrusion measuring 500 × 250 µm (width × height) extending from the top of the main electrode body. This protruding area, referred to as the “tab,” was utilized for imaging the electrode using limited field of view (FoV) techniques. Furthermore, an area of 5 mm × 10 mm was milled into the opposite side of the electrode to facilitate ultrasonic welding of an aluminium terminal onto the electrode's bare current collector.

The single‐layered pouch cell housed a NMC811 electrode, as previously described. To assemble the cell, the laser‐cut electrode was first ultrasonically welded to a positive aluminium terminal from MTI Corporation. Subsequently, it was heat‐sealed between two triple‐layered aluminium pouch cell materials. The assembly was then dried for 24 h at 80°C in a glass drying oven (B‐585, BUCHI Ltd) equipped with a ceramic‐reinforced tri‐layered polyolefin separator membrane (Celgard 2325, Celgard, LLC.) and a nickel negative electrode terminal from MTI Corp. Following the drying process, the pouch was transferred to an argon‐filled glove box for further assembly. Here, the lithium counter electrode from Goodfellows was positioned beneath the nickel terminal, after which two sides of the pouch were sealed using a vacuum sealing machine within the glove box. Before sealing the final edge of the pouch, an excess (200 µL) of 1.0 M lithium hexafluorophosphate in ethylene carbonate and ethyl methyl carbonate with 2% by weight of vinylene carbonate additive (1.0 M LiPF6 in EC:EMC (3:7 v/v) + 2% VC, Soulbrain MI) was added. The pouch cell was left undisturbed for 24 h to allow proper wetting of the electrodes by the electrolyte. Some gentle massaging of the cell was necessary during this time to aid in the distribution of the electrolyte.

The theoretical capacity of the cell was determined by calculating the product of the electrode's main body area (30 mm^2^) and the areal loading of the NMC811 electrode, assuming minimal contribution from the tab to the total capacity. For the cell containing a 2.2 mAh cm^−2^ electrode, the calculated theoretical capacity was 6.4 mAh. A low‐current potentiostat (SP‐300, Bio‐logic SAS) was utilized, employing a constant‐current constant‐voltage (CC‐CV) charge protocol. Each cell was charged to specified voltages (3.8, 4.0, 4.1, 4.2, 4.3, 4.4 vs Li/Li^+^) before discharging to the same voltages, see Figure A1. Charging and discharging were conducted at a C/3 rate based on the calculated theoretical capacity. Cells were allowed to rest once the current dropped below the C/20 threshold after the CV step, during which tomograms were obtained.

## Appendix B Effect of Lithiation on X‐ray Attenuation

Although the lithium content in the NMC811 structure varies during the experiment, the corresponding change in x‐ray transmission is negligible. Table B1 lists the mass attenuation coefficients and atomic weights for the constituent elements in Li_1_Ni_0.8_Mn_0.1_Co_0.1_O_2_ at an x‐ray energy of 27 keV (the average x‐ray energy in the operando experiment).

**Table B1:** Mass attenuation coefficients and atomic weights for elements in NMC at an x‐ray energy of 27 KeV.

Element	Li	Ni	Mn	Co	O
Mass attenuation coefficient (cm^2^ g^−1^)	0.00426	13.3	9.05	11.3	0.223
Atomic weight (g mol^−1^)	6.94	58.7	54.9	58.9	16.0

From the atomic weights and stoichiometry, the mass of each element per mole of Li_1_Ni_0.8_Mn_0.1_Co_0.1_O_2_ is:

(B1)
$$\begin{array}{cc} m_{\mathrm{Li}} & =1\times 6.94\;=\;6.94\;g\\ m_{\mathrm{Ni}} & =0.8\times 58.7=47.0\;g\\ m_{\mathrm{Mn}} & =0.1\times 54.9=5.49\;g\\ m_{\mathrm{Co}} & =0.1\times 58.9=5.89\;g\\ m_{O} & =2\times 16.0=32.0\;g \end{array}$$

The attenuation cross section for the delithiated compound Ni_0.8_Mn_0.1_Co_0.1_O_2_ is:

(B2)
$$\begin{array}{ccc} & & \Sigma_{delith}=\sum_{i}m_{i}\;\mu_{i}=47.0\times 13.3+5.49\times 9.05+5.89\times 11.3\\ & & +32.0\times 0.223=749\;{\mathrm{cm}}^{2}\;\left(3\;s.f.\right). \end{array}$$

Including lithium in Li_1_Ni_0.8_Mn_0.1_Co_0.1_O_2_ yields:

(B3)
$$\begin{array}{ccc} & & \Sigma_{lith}=6.94\times 0.00426+47.0\times 13.3+5.49\times 9.05+5.89\times 11.3\\ & & +32.0\times 0.223=749\;{\mathrm{cm}}^{2}\;\left(3\;s.f.\right). \end{array}$$

Hence, Σ_delith_ ≈ Σ_lith_, indicating that lithiation has a negligible effect on the x‐ray attenuation of NMC811 at 27 keV. This calculation assumes a monochromatic beam and neglects interatomic interactions, which is reasonable for illustrating the minimal impact of lithiation on x‐ray attenuation in this system.

## References

[1] H. D. Matthews , S. Wynes , Science 2022, 376, 1404. 35737785 10.1126/science.abo3378

[2] S.‐T. Myung , F. Maglia , K.‐J. Park , C. S. Yoon , P. Lamp , S.‐J. Kim , Y.‐K. Sun , ACS Energy Lett. 2017, 2, 196.

[3] R. Schmuch , R. Wagner , G. Hörpel , T. Placke , M. Winter , Nat. Energy 2018, 3, 267.

[4] F. Schipper , E. M. Erickson , C. Erk , J.‐Y. Shin , F. F. Chesneau , D. Aurbach , J. Electrochem. Soc. 2016, 164, A6220.

[5] E. A. Olivetti , G. Ceder , G. G. Gaustad , X. Fu , Joule 2017, 1, 229.

[6] S. Zheng , C. Hong , X. Guan , Y. Xiang , X. Liu , G.‐L. Xu , R. Liu , G. Zhong , F. Zheng , Y. Li , X. Zhang , Y. Ren , Z. Chen , K. Amine , Y. Yang , J. Power Sources 2019, 412, 336.

[7] F. Lin , I. M. Markus , D. Nordlund , T.‐C. Weng , M. D. Asta , H. L. Xin , M. M. Doeff , Nat. Commun. 2014, 5, 3529. 24670975 10.1038/ncomms4529

[8] J. Yang , Y. Xia , ACS Appl. Mater. Interfaces 2016, 8, 1297. 26695454 10.1021/acsami.5b09938

[9] H. Zheng , Q. Sun , G. Liu , X. Song , V. S. Battaglia , J. Power Sources 2012, 207, 134.

[10] D. R. Gallus , R. Schmitz , R. Wagner , B. Hoffmann , S. Nowak , I. Cekic‐Laskovic , R. W. Schmitz , M. Winter , Electrochim. Acta 2014, 134, 393.

[11] D. Streich , C. Erk , A. Guéguen , P. Muüller , F.‐F. Chesneau , E. J. Berg , J. Phys. Chem. C 2017, 121, 13481.

[12] N. Laszczynski , S. Solchenbach , H. A. Gasteiger , B. L. Lucht , J. Electrochem. Soc. 2019, 166, A1853.

[13] A. O. Kondrakov , A. Schmidt , J. Xu , H. Geßwein , R. Möunig , P. Hartmann , H. Sommer , T. Brezesinski , J. Janek , J. Phys. Chem. C 2017, 121, 3286.

[14] P. Yan , J. Zheng , M. Gu , J. Xiao , J.‐G. Zhang , C.‐M. Wang , Nat. Commun. 2017, 8, 14101. 28091602 10.1038/ncomms14101 PMC5241805

[15] H.‐H. Sun , A. Manthiram , Chem. Mater. 2017, 29, 8486.

[16] P. Li , Y. Zhao , Y. Shen , S.‐H. Bo , J. Phys.: Energy 2020, 2, 022002.

[17] C. R. Birkl , M. R. Roberts , E. McTurk , P. G. Bruce , D. A. Howey , J. Power Sources 2017, 341, 373.

[18] H. C. Parks , A. M. Boyce , A. Wade , T. M. Heenan , C. Tan , E. Martínez‐Pañeda , P. R. Shearing , D. J. Brett , R. Jervis , J. Mater. Chem. A 2023, 11, 21322.

[19] H. Parks , NMC811 4D Nanotomography Tiff Stacks 2023, 10.5522/04/22120061.v1.

[20] T. M. Heenan , C. Tan , J. Hack , D. J. Brett , P. R. Shearing , Mater. Today 2019, 31, 69.

[21] Y.‐c. K. Chen‐Wiegart , Z. Liu , K. T. Faber , S. A. Barnett , J. Wang , Electrochem. Commun. 2013, 28, 127.

[22] R. Moroni , M. Börner , L. Zielke , M. Schroeder , S. Nowak , M. Winter , I. Manke , R. Zengerle , S. Thiele , Sci. Rep. 2016, 6, 30109. 27456201 10.1038/srep30109 PMC4960488

[23] C. Xu , K. Märker , J. Lee , A. Mahadevegowda , P. J. Reeves , S. J. Day , M. F. Groh , S. P. Emge , C. Ducati , B. Layla Mehdi , C. C. Tang , C. P. Grey , Nat. Mater. 2021, 20, 84. 32839589 10.1038/s41563-020-0767-8

[24] L. Petrich , D. Westhoff , J. Feinauer , D. P. Finegan , S. R. Daemi , P. R. Shearing , V. Schmidt , Comput. Mater. Sci. 2017, 136, 297.

[25] O. Badmos , A. Kopp , T. Bernthaler , G. Schneider , J. Intell. Manuf. 2020, 31, 885.

[26] D. Westhoff , D. P. Finegan , P. R. Shearing , V. Schmidt , J. Microsc. 2018, 270, 71. 29071715 10.1111/jmi.12653

[27] Y. Yang , R. Xu , K. Zhang , S.‐J. Lee , L. Mu , P. Liu , C. K. Waters , S. Spence , Z. Xu , C. Wei , D. J. Kautz , Q. Yuan , Y. Dong , Y.‐S. Yu , X. Xiao , Y. Dong , Y.‐S. Yu , X. Xiao , H.‐K. Lee , P. Pianetta , P. Cloetens , J.‐S. Lee , K. Zhao , F. Lin , Y. Liu , Adv. Energy Mater. 2019, 9, 1900674.

[28] S. R. Daemi , C. Tan , T. G. Tranter , T. M. Heenan , A. Wade , L. Salinas‐Farran , A. V. Llewellyn , X. Lu , A. Matruglio , D. J. Brett , R. Jervis , Small Methods 2022, 6, 2200887. 10.1002/smtd.202200887 36089665

[29] A. Wade , T. Heenan , M. Kok , T. Tranter , A. Leach , C. Tan , R. Jervis , D. Brett , P. Shearing , npj Mater. Degrad. 2022, 6, 44.

[30] T. M. Heenan , A. V. Llewellyn , A. S. Leach , M. D. Kok , C. Tan , R. Jervis , D. J. Brett , P. R. Shearing , Adv. Sci. 2020, 7, 2000362. 10.1002/advs.202000362 PMC7312274 32596123

[31] C. Tan , S. Daemi , T. Heenan , F. Iacoviello , A. Leach , L. Rasha , R. Jervis , D. Brett , P. Shearing , J. Electrochem. Soc. 2020, 167, 060512.

[32] C. Rau , A. Bodey , M. Storm , S. Cipiccia , S. Marathe , M.‐C. Zdora , I. Zanette , U. Wagner , D. Batey , X. Shi , Micro‐and nano‐tomography at the DIAMOND beamline I13L imaging and coherence, in *Developments in X‐Ray Tomography XI* , vol. 10391, SPIE, Bellingham, WA 2017, pp. 134–141.

[33] S. Van der Walt , J. L. Schönberger , J. Nunez‐Iglesias , F. Boulogne , J. D. Warner , N. Yager , E. Gouillart , T. Yu , PeerJ 2014, 2, e453. 25024921 10.7717/peerj.453 PMC4081273

[34] R. C. Atwood , A. J. Bodey , S. W. Price , M. Basham , M. Drakopoulos , Philos. Trans. R. Soc., A 2015, 373, 20140398. 10.1098/rsta.2014.0398 PMC4424489 25939626

[35] A. Bodey , C. Rau , in Journal of Physics: Conference Series, vol. 849, IOP Publishing, Bristol 2017, p. 012038.

[36] N. T. Vo , R. C. Atwood , M. Drakopoulos , Opt. Express 2018, 26, 28396. 30470012 10.1364/OE.26.028396

[37] D. Gürsoy , F. De Carlo , X. Xiao , C. Jacobsen , J. Synchrotron Radiat. 2014, 21, 1188. 25178011 10.1107/S1600577514013939 PMC4181643

[38] B. C. Lowekamp , D. T. Chen , L. Ibáñez , D. Blezek , Front. Neuroinform. 2013, 7, 45. 24416015 10.3389/fninf.2013.00045 PMC3874546

[39] W. Silversmith , P. Hilei , seung‐lab/euclidean‐distance‐transform‐3d: Zenodo Release , 10.5281/zenodo.10815871, (accessed: March 2024).

[40] A. M. Boyce , E. Martínez‐Pañeda , A. Wade , Y. S. Zhang , J. J. Bailey , T. M. Heenan , D. J. Brett , P. R. Shearing , J. Power Sources 2022, 526, 231119.

[41] T. M. M. Heenan , A. Wade , C. Tan , J. E. Parker , D. Matras , A. S. Leach , J. B. Robinson , A. Llewellyn , A. Dimitrijevic , R. Jervis , P. D. Quinn , D. J. L. Brett , P. R. Shearing , Adv. Energy Mater. 2020, 10, 2002655.

[42] A. V. Llewellyn , A. S. Leach , I. Mombrini , A. Matruglio , J. Diao , C. Tan , T. M. M. Heenan , I. K. Robinson , D. Brett , R. Jervis , P. R. Shearing , in Electrochemical Society Meeting Abstracts 241, 2, The Electrochemical Society, Inc. , New Jersey 2022, pp. 177–177.

[43] H. C. W. Parks , M. P. Jones , A. Wade , A. V. Llewellyn , C. Tan , H. T. Reid , R. Zieche , T. M. M. Heenan , S. Marathe , C. Rau , P. R. Shearing , R. Jervis , Non-linear cracking response to voltage revealed by operando X-ray tomography in polycrystalline NMC811’, EES Batteries 2024, 1, 482.

[44] C. Xu , A. J. Merryweather , S. S. Pandurangi , Z. Lun , D. S. Hall , V. S. Deshpande , N. A. Fleck , C. Schnedermann , A. Rao , C. P. Grey , Joule 2022, 6, 2535.

[45] H. Liu , J. M. Foster , A. Gully , S. Krachkovskiy , M. Jiang , Y. Wu , X. Yang , B. Protas , G. R. Goward , G. A. Botton , J. Power Sources 2016, 306, 300.

[46] S. Bessette , A. Paolella , C. Kim , W. Zhu , P. Hovington , R. Gauvin , K. Zaghib , Sci. Rep. 2018, 8, 17575. 30514866 10.1038/s41598-018-33608-3 PMC6279772

[47] D. Hou , Z. Xu , Z. Yang , C. Kuai , Z. Du , C.‐J. Sun , Y. Ren , J. Liu , X. Xiao , F. Lin , Nat. Commun. 2022, 13, 3437. 35705552 10.1038/s41467-022-30935-y PMC9200779

[48] C. Cao , M. F. Toney , T.‐K. Sham , R. Harder , P. R. Shearing , X. Xiao , J. Wang , Mater. Today 2020, 34, 132.

[49] Y. Zhu , N. Samadi , M. Martinson , B. Bassey , Z. Wei , G. Belev , D. Chapman , Phys. Med. Biol. 2014, 59, 2485. 24778351 10.1088/0031-9155/59/10/2485

[50] A. Beale , S. Jacques , M. Di Michiel , J. Mosselmans , S. Price , P. Senecal , A. Vamvakeros , J. Paterson , Philos. Trans. R. Soc. A Math. Phys. Eng. Sci. 2017, 376, 20170057. 10.1098/rsta.2017.0057 PMC5719219 29175905

[51] D. Matras , A. Vamvakeros , S. Jacques , M. Michiel , V. Middelkoop , Z. Ismagilov , E. Matus , V. Kuznetsov , R. Cernik , A. Beale , J. Mater. Chem. A 2021, 9, 11331.

[52] H. Parks , M. Jones , R. Jervis , UCL Research Data Repository 2024.

